# FERMT3 mediates cigarette smoke-induced epithelial–mesenchymal transition through Wnt/β-catenin signaling

**DOI:** 10.1186/s12931-021-01881-y

**Published:** 2021-11-06

**Authors:** Xiaoshan Su, Junjie Chen, Xiaoping Lin, Xiaoyang Chen, Zhixing Zhu, Weijing Wu, Hai Lin, Jianming Wang, Xiangjia Ye, Yiming Zeng

**Affiliations:** 1 Department of Pulmonary and Critical Care Medicine, The Second Affiliated Hospital of Fujian Medical University, Respirology Medicine Centre of Fujian Province, Quanzhou, China; 2 Department of Pulmonary and Critical Care Medicine, The First Affiliated Hospital of Wenzhou Medical University, Wenzhou, China; 3 Department of Critical Care Medicine, Quanzhou First Hospital Affiliated to Fujian Medical University, Quanzhou, China

**Keywords:** FERMT3, EMT, COPD, Wnt/β-catenin

## Abstract

**Background:**

Cigarette smoking is a major risk factor for chronic obstructive pulmonary disease (COPD) and lung cancer. Epithelial–mesenchymal transition (EMT) is an essential pathophysiological process in COPD and plays an important role in airway remodeling, fibrosis, and malignant transformation of COPD. Previous studies have indicated FERMT3 is downregulated and plays a tumor-suppressive role in lung cancer. However, the role of FERMT3 in COPD, including EMT, has not yet been investigated.

**Methods:**

The present study aimed to explore the potential role of FERMT3 in COPD and its underlying molecular mechanisms. Three GEO datasets were utilized to analyse FERMT3 gene expression profiles in COPD. We then established EMT animal models and cell models through cigarette smoke (CS) or cigarette smoke extract (CSE) exposure to detect the expression of FERMT3 and EMT markers. RT-PCR, western blot, immunohistochemical, cell migration, and cell cycle were employed to investigate the potential regulatory effect of FERMT3 in CSE-induced EMT.

**Results:**

Based on Gene Expression Omnibus (GEO) data set analysis, FERMT3 expression in bronchoalveolar lavage fluid was lower in COPD smokers than in non-smokers or smokers. Moreover, FERMT3 expression was significantly down-regulated in lung tissues of COPD GOLD 4 patients compared with the control group. Cigarette smoke exposure reduced the FERMT3 expression and induces EMT both in vivo and in vitro. The results showed that overexpression of FERMT3 could inhibit EMT induced by CSE in A549 cells. Furthermore, the CSE-induced cell migration and cell cycle progression were reversed by FERMT3 overexpression. Mechanistically, our study showed that overexpression of FERMT3 inhibited CSE-induced EMT through the Wnt/β-catenin signaling.

**Conclusions:**

In summary, these data suggest FERMT3 regulates cigarette smoke-induced epithelial–mesenchymal transition through Wnt/β-catenin signaling. These findings indicated that FERMT3 was correlated with the development of COPD and may serve as a potential target for both COPD and lung cancer.

**Supplementary Information:**

The online version contains supplementary material available at 10.1186/s12931-021-01881-y.

## Introduction

Chronic obstructive pulmonary disease (COPD) is a chronic progressive lung disease that is usually caused by exposure to harmful gases or particles [1]. The pathological features of COPD include inflammation, airway and parenchymal remodeling, small airway fibrosis and obliteration, and it is also associated with an increased risk for developing lung cancer [2]. Moreover, epidemiological evidence shows that COPD is a major independent risk factor for lung cancer in smokers, increasing the risk of developing lung cancer up to 4.5-fold [3, 4]. Indeed, COPD and lung cancer have common multiple biological mechanisms, including Extracellular Matrix destruction, chronic inflammation, cell proliferation, aberrant wound repair, and epithelial–mesenchymal transition (EMT) [5, 6]. EMT is a physiological and pathological phenomenon in which epithelial cells lose polarity and transform into mesenchymal cells. Numerous studies indicated that activation of MET was observed in smokers and particularly in COPD smokers, playing an important role in airway remodeling, fibrosis and malignant transformation [7, 8]. Additionally, major signaling pathways such as Wnt/β-catenin and transforming growth factor-β (TGF-β)/Smad are involved in EMT in COPD [5, 9, 10]. However, the mechanism of EMT in COPD is not fully understood.

FERMT3, also known as URP-2 and kindlin-3, belongs to the kindlin family of binding proteins containing the FERM domain and consists of three members (FERMT1–3). Kindlins play a crucial role in various biological activities, including integrin activation, cell adhesion, migration, cell–cell junctions, and differentiation [11]. So far, studies have shown that FERMT3 plays crucial roles in important physiological and pathological processes, including integrin activation-mediated signaling, cell adhesion and migration, immune regulation, and cancer progression [12–14]. It was found that FERMT3 expression was down-regulated in lung cancer, and depletion of FERMT3 enhances cell proliferation, migration, and invasion in lung cancer cells [13, 15]. Nevertheless, the role of FERMT3 in EMT and COPD has not been reported.

In this work, Gene Expression Omnibus (GEO) dataset analysis demonstrated that FERMT3 expression in COPD smokers was lower than that in non-smokers or smokers, and FERMT3 was significantly down-regulated in lung tissues of COPD GOLD 4 patients compared with the control group. Moreover, cigarette smoke exposure downregulated the FERMT3 expression and induces EMT both in vivo and in vitro. Mechanistically, overexpression of FERMT3 inhibited cigarette smoke extract (CSE)-induced EMT through blocking the Wnt/β-catenin pathway. These findings provide new insights into the basis of EMT and suggest FERMT3 may serve as a potential target for both COPD and lung cancer.

## Materials and methods

### Data acquisition

The published microarray datasets, GSE130928 (including 24 non-smoker, 42 smoker, and 22 COPD-smoker), GSE13896 (containing 24 non-smoker, 34 smoker, and 12 COPD-smoker), and GSE151052 (containing 77 COPD samples and 40 control samples) were retrieved from the NCBI public database. Alveolar macrophages isolated from the bronchoalveolar lavage fluid (BALF) of subjects with COPD-smoker and healthy non-smoker controls in GSE130928 and GSE13896. The GSE151052 provided 117 lung tissue samples from 10 explanted lungs of patients with very severe (Global Initiative for Obstructive Lung Disease, GOLD 4) COPD treated by lung transplantation, and 5 unused donor control lungs (8/lung, three samples were excluded due to quality control). The characteristics of study individuals were summarized in Additional file 1: Tables S1–S3. Differential expression of FERMT3 in Non-smoker and COPD-smoker was analyzed using the GEO2R online analysis tool (http://www.ncbi.nlm.nih.gov/geo/geo2r).

### Animals exposure

C57BL/6 mice (male, 6–8 weeks) were purchased from Shanghai Animal Laboratory Center (Shanghai, China) and raised in the Laboratory Animal Center of Quanzhou Medical College. Twenty mice were divided into two groups of 10 mice each: control group (control mice) and cigarette smoke exposure group (CS-exposed mice). We designed a custom-made smoking chamber (70 cm × 50 cm × 40 cm) to establish the CS exposure model. In this work, CS-exposed mice were placed in the chamber and exposed whole body to CS, as described before [16]. Briefly, the mice were exposed to six cigarettes (containing 0.8 mg of nicotine and 10 mg of tar per cigarette, Shishi, China) 3 times a day, 6 days/week for a total of 12 weeks. The control group was exposed to filtered air. After the last exposure, all the mice were sacrificed.

### Cigarette smoke extract preparation

Cigarette smoke extract (CSE) was prepared as described below. In short, two non-filtered cigarettes (containing 0.8 mg of nicotine and 10 mg of tar per cigarette, Shishi, China) were burned with a vacuum pump, and then smoke was bubbled through the 20 ml serum-free medium to collect CSE. The pH of CSE was adjusted to 7.4 and filtered through a 0.22 μm pore filter. CSE was standardized by measuring the absorbance at a wavelength of 340 nm. The final solution was considered as 100% CSE. Then the CSE solutions were sub-packed and stored at − 80 °C.

### Pulmonary function test

Pulmonary function tests on mice were measured via a pulmonary function testing system (Buxco Research Systems, USA) according to the manufacturer’s instructions. Briefly, mice were anesthetized by intraperitoneal injection of pentobarbital (10 μl of 1% pentobarbital/g body weight). After deep anesthesia, mice were tracheostomized and then placed into the body chamber of the system. The trachea was connected with the pipe of the system and ventilated in order to perform the pulmonary function test. Parameters of pulmonary function included the ratios of forced expiratory volume (FEV) at 0.2 s (FEV0.2) to forced vital capacity (FEV0.2%), functional residual capacity (FRC), and inspiratory resistance (RI).

### Cell culture

The human type II alveolar cells A549 and human bronchial epithelioid cells HBE were obtained from Procell life science and Technology Co., Ltd (Wuhan, China). The cells were cultured in DMEM and RPMI-1640 medium (Gibico) supplemented with 10% fetal bovine serum (FBS, Gibico), penicillin (100 U/ml), and streptomycin (100 U/ml). Cells were grown in a humidified incubator at 37 °C and 5% CO_2_.

### CCK-8 cell viability assay

Cell viability assays were performed using the Cell Counting Kit-8 (CCK-8) (Meilun Biotechnology, Dalian, China). A549 cells and HBE cells were treated with different concentrations of CSE for 0, 12, 24, and 48 h, respectively. At each time point, 10 µl of CCK-8 solution was added and the cells were incubated at 37 °C for 1 h. Then, the optical density was measured at 450 nm using a microplate reader (Thermo Fisher Scientific). Cell viability (%) = [(As − Ab)/(Ac − Ab)] × 100; where As = absorbance of the experimental well, Ab = blank well absorbance and Ac = control well absorbance.

### Reagents

Anti-FERMT3 (ab68040), anti-E-cadherin (ab40772), anti-Vimentin (ab92547) and anti-Snail (ab216347) were purchased from Abcam (Abcam, USA). Anti-GAPDH (AB0037) was purchased from Abways Biotechnology (Shanghai, China). Anti-β-catenin (#8480), anti-p-β-catenin (#5651), anti-GSK-3β (#12456), anti-GSK-3β (#5558) was purchased from Cell Signaling Technology. Lithium Chloride (LiCl), an activator of the Wnt/β-catenin signaling pathway, was purchased from Sigma Aldrich.

### Cell transfection

Control vector (pEX-3 plasmid), FERMT3 vector (recombinant plasmid containing FERMT3, pEX-3-FERMT3), control siRNA (Si-NC), and FERMT3 siRNA (Si-FERMT3) were obtained from GenePharma (Shanghai, China). The vector and siRNA sequences were transfected into A549 cells, respectively. The siRNA sequences were designed and synthesized by GenePharma (Shanghai, China). When cells up to 60–70% confluence, A549 cells were transfected with vector or siRNA using Lipofectamine 3000 (Invitrogen, USA). The relevant sequences are as below:

Si-FERMT3 forward, 5ʹ-GCUUCAAGUACUACAGCUUTT-3ʹ;

Si-FERMT3 reverse, 3ʹAAGCUGUAGACUUGAAGCTT-5ʹ;

Si-NC forward, 5ʹ-UUCUUCCGAACGUGUCACGUTT-3ʹ;

Si-NC reverse, 3ʹACGUGACACGUUCGGAGAATT-5ʹ.

### RNA extraction and quantitative real-time PCR (qRT-PCR)

Total RNA was isolated using Trizol reagent (Invitrogen) and reversely transcribed into cDNA using the PrimeScript™ RT reagent kit (Takara). PCR amplification was conducted using the SYBR Green PCR kit (Takara) in a 7500 Real-Time PCR System (Thermo Fisher Scientific) and GAPDH was used as the internal control. The relative expression levels were analyzed by the 2^−ΔΔCT^ method. The PCR primers sequences were used as below:

FERMT3 forward, 5′-ACTGCACCGAGGAGGAGATGATG-3′

FERMT3 reverse, 5′-CCTTGAGGTTGAGCTGCTGAATGG-3′

GAPDH forward, 5′-CTCCTGCACCACCAACTGCTTAG-3′

GAPDH reverse, 5′-GACGCCTGCTTCACCACCTTC-3′

### Western blot assays

Total tissues and cell proteins were isolated using RIPA lysis buffer (Beyotime) and the protein concentration was measured using BCA protein assay (Suolabao). Protein samples were boiled in a 4× sample loading buffer for 10 min. All samples were subjected to electrophoresis and electrically transferred to the PDVF membrane (Millipore). The membranes were blocked with 5% skim milk solution for 2 h and incubated overnight with primary antibody GAPDH, FERMT3, E-cadherin, Vimentin, β-catenin, p-β-catenin, GSK-3β, and p-GSK-3β at 4 °C, respectively. Next, the primary antibodies were washed and the membrane was incubated with the secondary antibodies (Beyotime) for 1 h. Proteins were quantified using ImageJ software with GAPDH as the endogenous reference.

### Histology and immunohistochemistry

Lung tissues were fixed with neutral-buffered formalin overnight and embedded in paraffin. Lung sections were stained with standard hematoxylin–eosin staining (H&E) for histological assessment. Immunohistochemical staining was applied to detect the protein expression of FERMT3, as described previously [17]. Briefly, sections of lung tissue were deparaffinized with xylene and rehydrated with gradient ethanol. Then, the endogenous peroxide activity was blocked with 3% hydrogen peroxide. After blocking with serum, the sections were incubated with FERMT3 primary antibodies (1:250; Abcam) for 2 h at room temperature. These sections were then incubated with horseradish-peroxidase conjugated anti-rabbit IgG antibody at room temperature for 30 min and developed using DAB. Staining was quantified using Image-Pro 6.1 software.

### Cell migration assay

Migration was evaluated by wound-healing and transwell assays. For wound-healing experiments, A549 cells were seeded to at least 90% fusion in 12-well plates and then scratched by a 10 ul pipette tip. Migrating cells were observed under a microscope at 0 h and 24 h, respectively. The scratch area is quantified by ImageJ. Transwell (CoStar #3422) with an 8.0 μm aperture of 24 Wells was used for Transwell cell migration assay. Briefly, 200 μl of serum-free medium (1 × 106 cells) was added to the upper transwell chamber, and 500 μl of medium containing 20% FBS was placed into the lower chamber. After 24 h, the cells were fixed in 4% paraformaldehyde for 30 min, stained with 1% crystal violet for 30 min, and counted.

### Cell cycle

Cell cycle was detected through flow cytometry through the Cell Cycle Analysis Kit (Beyotime). In brief, differentially treated cells (1.0 × 106) were collected, fixed in 70% cold ethanol, and stored at 4 °C for 2 h. Then, 25 μl propidium iodide and 10 μl of RNase were added into cells and incubated at 37 °C for 30 min. After incubation in the dark, the cell cycle distribution was detected by flow cytometry. Cell distribution in the G1, S, and G2/M phases was analyzed using Modfit software. These experiments were performed in triplicate.

### Statistical analysis

Data were expressed as means ± SEM and analyzed with the help of GraphPad Prism (version 8.0). One-way ANOVA analysis of variance was applied for multiple comparisons. Student’s t-test was used in only two groups comparison. P < 0.05 was considered statistically significant.

## Results

### FERMT3 expression was downregulated in COPD patients, CS exposed mice, and CES-induced A549 cells

**Fig. 1:**
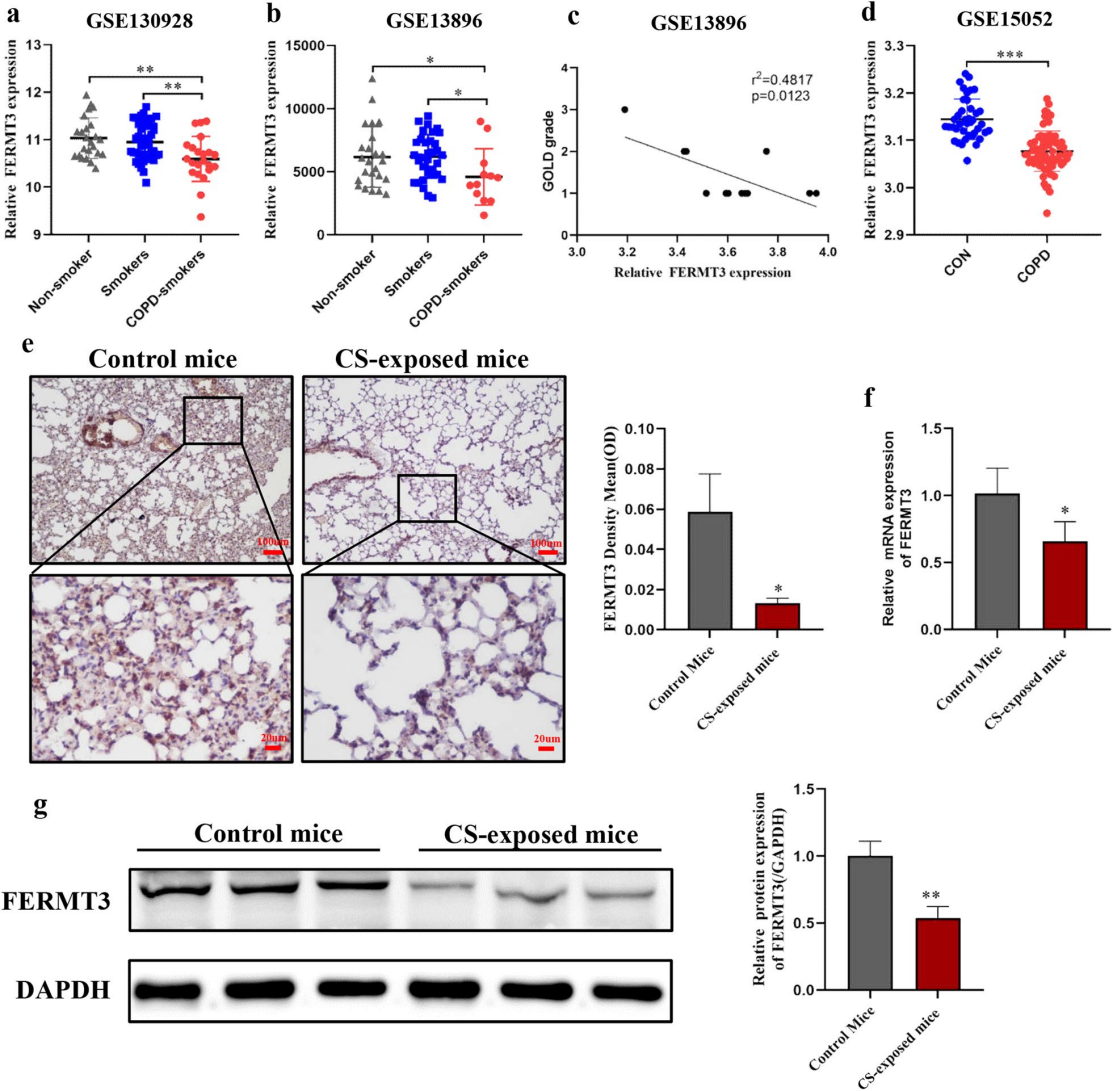
FERMT3 was downregulated observed in COPD patients and CS-exposed mice. **a**, **b** FERMT3 expression levels in BALF of non-smoker, smoker, and COPD-smoker in GSE130928 and GSE13896. **c** Correlations between the GOLD grade groups and expression levels of FERMT3 in GSE13896. **d** FERMT3 expression levels in lung tissues of COPD GOLD 4 patients and controls in GSE151052. **e** The protein expression of FERMT3 in lung tissues from the control mice and COPD mice via immunohistochemistry (× 100 magnification; × 400 magnification). n = 5 mice/per group. **f** PCR analysis of FERMT3 mRNA levels in lung tissues of control mice and COPD mice. n = 6 mice/per group. **g** Western blotting analysis of FERMT3 protein levels in lung tissues of control mice and COPD mice. n = 6 mice/per group. *P < 0.05, **P < 0.01, compared with control

Firstly, we explored the expression level of FERMT3 in COPD in the three GEO datasets. As shown in Fig. 1a, b, the expression of FERMT3 in BALF of COPD-smokers was significantly lower than that of non-smokers or smokers in GSE130928 and GSE13896. In addition, according to the classification of GOLD grade, there was a significant difference in the FERMT3 level among different GOLD grade groups (Fig. 1c). As the available number of GOLD patients is small (n = 12), further studies with larger sample sizes are required to validate the correlation between FERMT3 level and lung function. GSE151052 database analysis further confirmed that FERMT3 expression was significantly down-regulated in lung tissues of COPD GOLD 4 patients compared with the control group (Fig. 1d).

Next, we investigated whether FERMT3 expression was similarly altered in CS-exposed mice. Compared with the control mice, CS-exposed mice lung showed damage parenchymal alveolar destruction and developed chronic neutrophilic inflammation (Additional file 2: Fig. S1a). Furthermore, we determined the pulmonary function of mice to obtain more accurate data. Compared with the control mice, FEV0.2/FVC was significantly lower in CS-exposed mice (Additional file 2: Fig. S1b). Besides, the RI level was higher in CS-exposed mice (Additional file 2: Fig. S1c). But there was no significance in FRC between CS-exposed mice and control mice (Additional file 2: Fig. S1d). These results suggest that the CS-exposed mice model of COPD was constructed successfully. Consistent with our findings in patients with COPD, FERMT3 was downregulated at both mRNA and protein levels in CS-exposed mice compared to control mice (Fig. 1e–g).

**Fig. 2:**
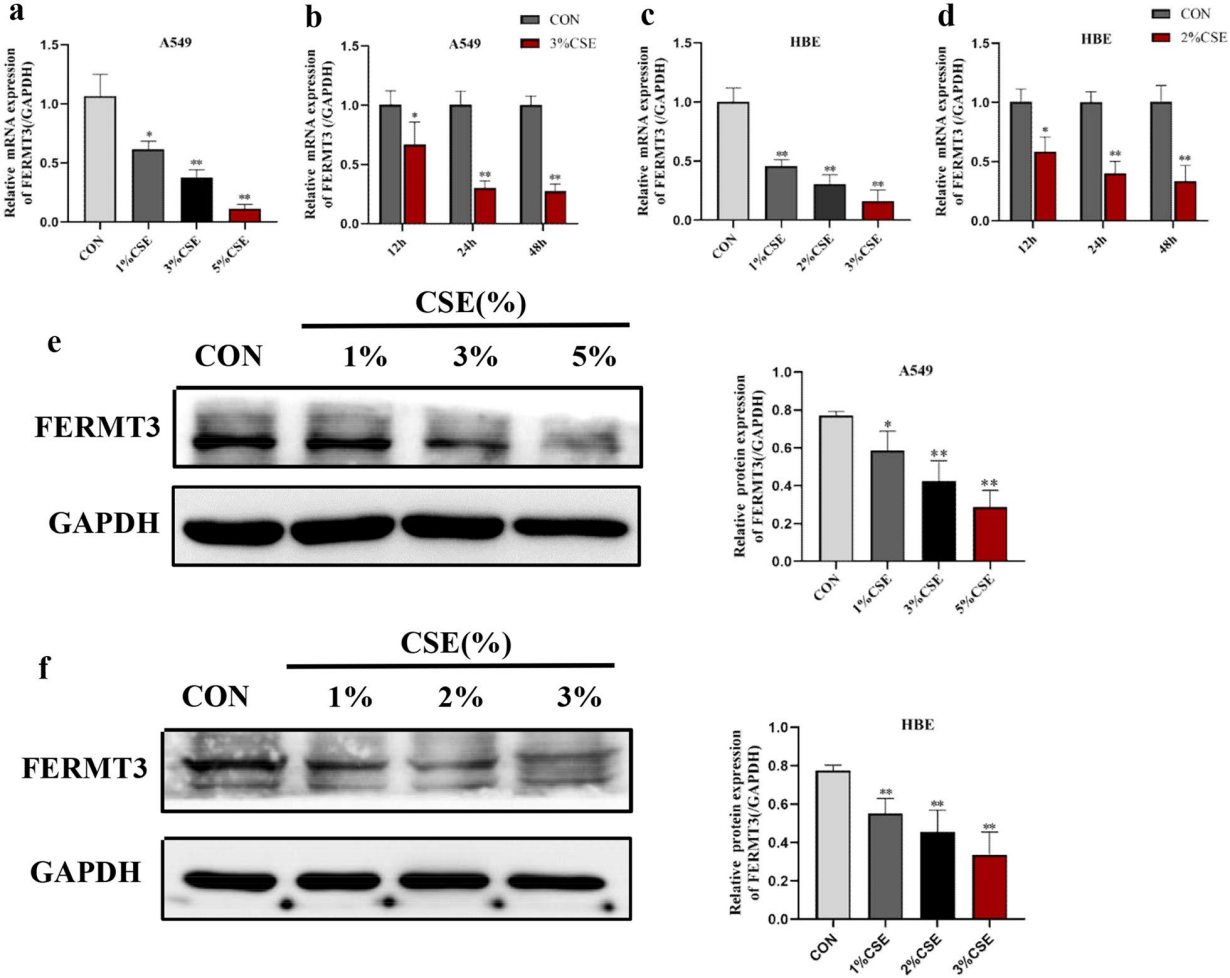
FERMT3 was downregulated observed in CES-induced A549 cells and HBE cells. **a**, **b** PCR showed the effect of CSE (0%, 1%, 3%, and 5%) and 3% CSE on FERMT3 mRNA in A549 cells at different time (12, 24, and 48 h). **c**, **d** PCR showed the effect of CSE (0%, 1%, 2%, and 3%) and 2% CSE on FERMT3 mRNA in HBE cells at different time (12, 24, and 48 h). **e**, **f** CSE downregulated the FERMT3 protein expression in a concentration-dependent manner in A549 cells and HBE cells. n = 3 per group. *P < 0.05, **P < 0.01, compared with control

Subsequently, human type II alveolar cells (A549) and human bronchial epithelioid cells (HBE) were exposed to CSE. We first assessed the cytotoxicity of CSE with the CCK-8 kit. Different concentrations of the CSE and different time points (0, 12, 24, and 48 h) were tested. As the exposure dose and time increased, the cell viability decreased. Thus, CSE affected cell viability in a dose-dependent and time-dependent manner (Additional file 3: Fig. S2a, b). We then measured the effect of different concentrations and time points of CSE exposure on FERMT3 expression. PCR demonstrated that the CSE treatment significantly reduced the levels of FERMT3 mRNA expression in a dose- and time-dependent manner in both A549 cells and HBE cells (Fig. 2a–d). Moreover, Western blot confirmed that CSE exposure resulted in a dose-dependent loss of FERMT3 protein (Fig. 2e–f). To investigate the role of FERMT3 in CSE-induced in alveolar type II epithelium, we used A549 as a cell model for subsequent studies.

Taken together, the above results suggest that FERMT3 was significantly downregulated in COPD and might play an important role in the development and progression of COPD.

### Cigarette smoke-induced EMT in vitro and in vivo

**Fig. 3:**
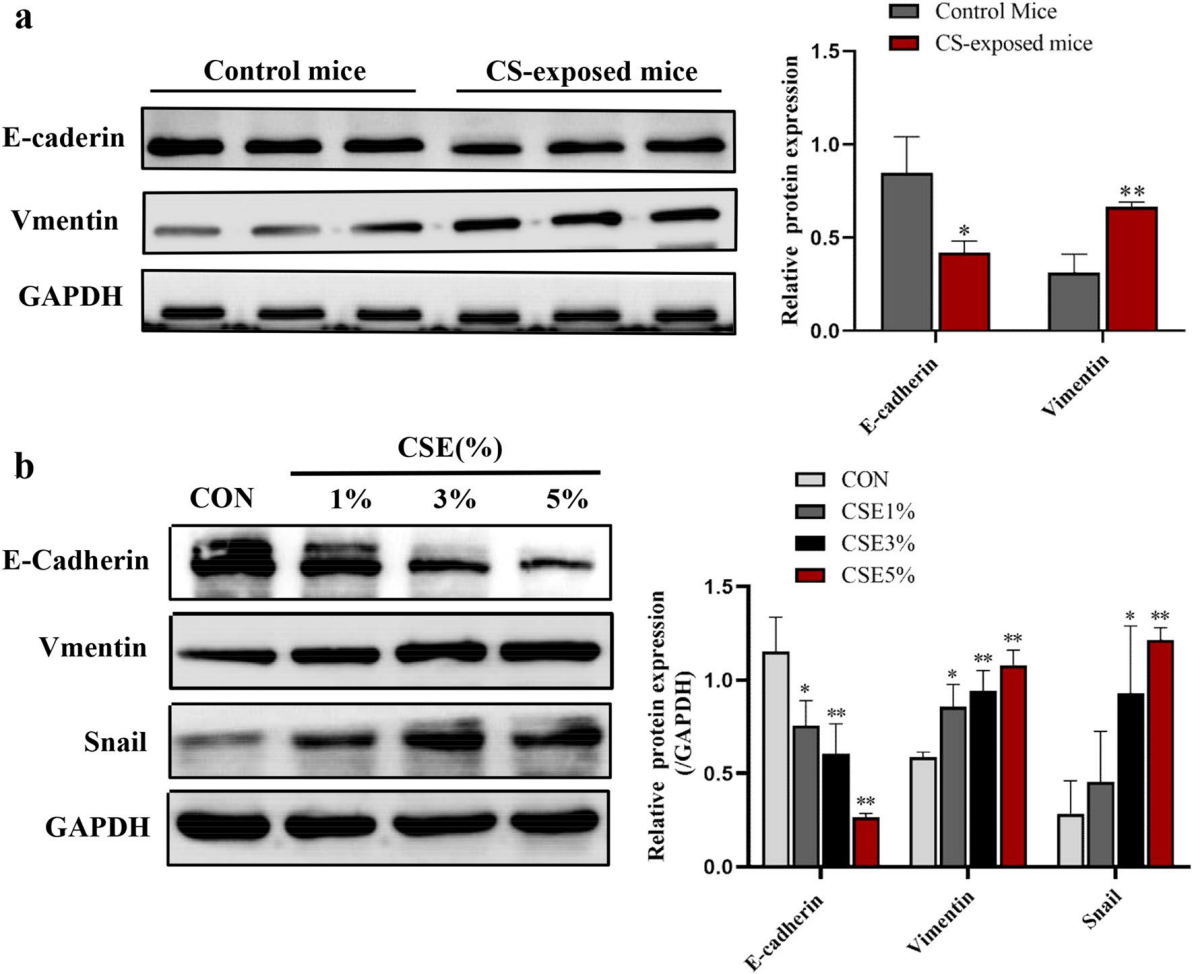
Cigarette smoke-induced EMT in mice and A549 cells. **a** E-cadherin and Vimentin in lung tissues from mice exposed to CS or control air. n = 3 per group. **b** E-cadherin, Vimentin, and Snail protein expression in A549 cells treated by CSE in a series of concentrations CSE for 24 h. n = 3 per group. *P < 0.05, **P < 0.01, compared with control

Accumulating studies have shown that Cigarette smoke induces epithelial–mesenchymal transition in COPD [16, 18, 19]. To verify that cigarette smoke induces the occurrence of EMT, E-cadherin and Vimentin serve as markers for epithelial cells and mesenchymal cells, respectively. After 12 weeks of cigarette smoke exposure, the protein expression of E-cadherin was downregulated while Vimentin was upregulated significantly in CS-exposed mice (Fig. 3a). Moreover, A549 cells were treated with 0, 1%, 3% and 5% CSE for 24 h, respectively. The data showed that CSE exposure resulted in a dose-dependent loss of E-cadherin protein, with a concomitant gain in Vimentin (Fig. 3b). In addition, CSE exposure also increased Snail protein, an EMT-inducing transcriptional factor that plays a critical role in the EMT process.

### FERMT3 regulated cigarette smoke-induced EMT in A549 cells

**Fig. 4:**
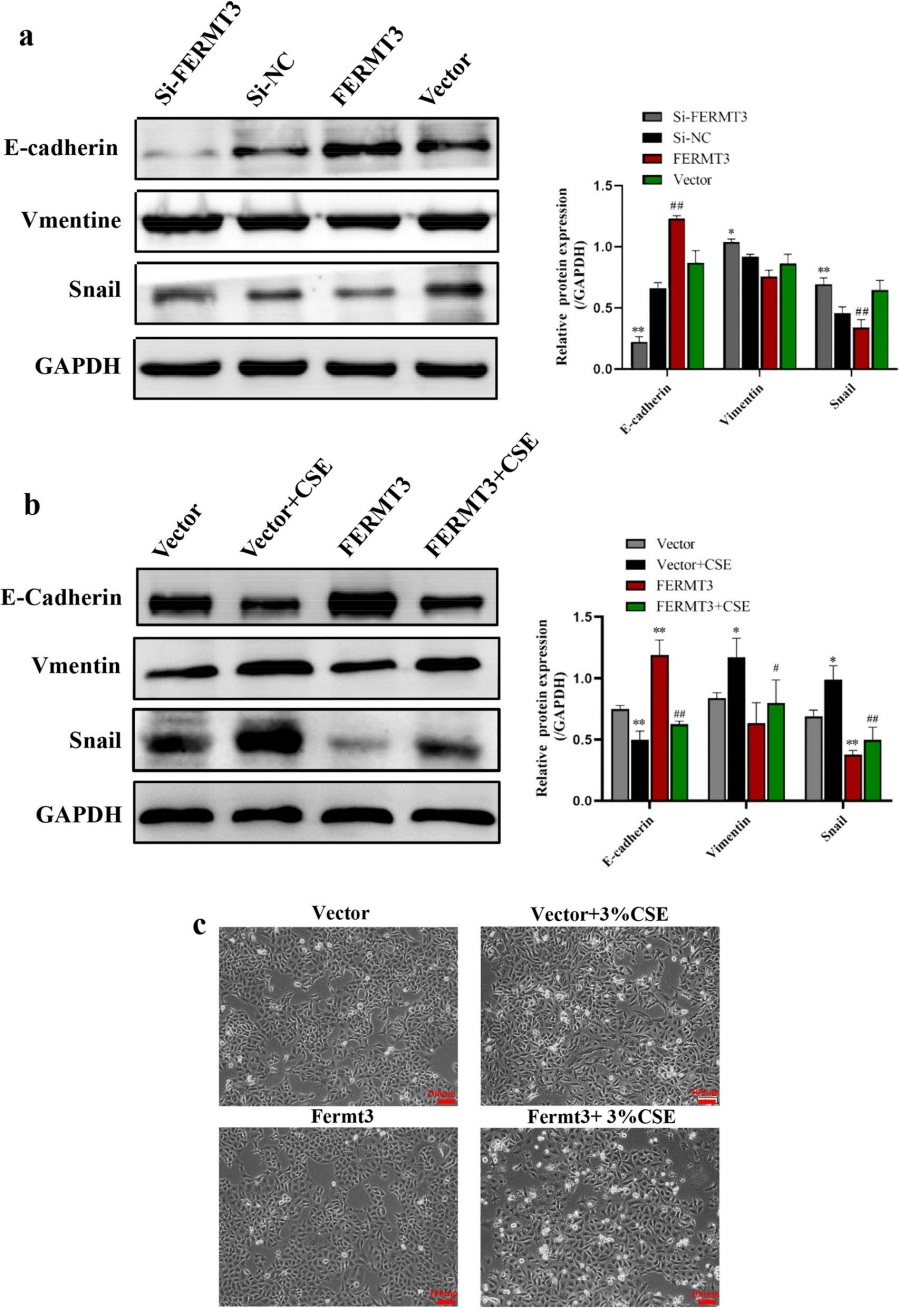
The effect of FERMT3 on CSE induced EMT in A549 cells. **a** Ecadherin, Vimentin and Snail protein expression in A549 cells transfected with Si-NC, Si-FERMT3, FERMT3 vector or control vector for 24 h. *P < 0.01, **P < 0.01, compared with Si-NC; ^#^P < 0.05, ^##^P < 0.01, compared with control vector. n = 3 per group. **b** Ecadherin, Vimentin and Snail protein expression in A549 cells transfected with control vector or FERMT3 vector and then treated by 3% CSE for 24 h. *P < 0.01, **P < 0.01, compared with control vector; ^#^P < 0.05, ^##^P < 0.01, compared with control vector + 3% CSE. n = 3 per group. **c** Morphological changes of CSE-treated A549 cells after transfected with control vector or FERMT3 vector were observed

**Fig. 5:**
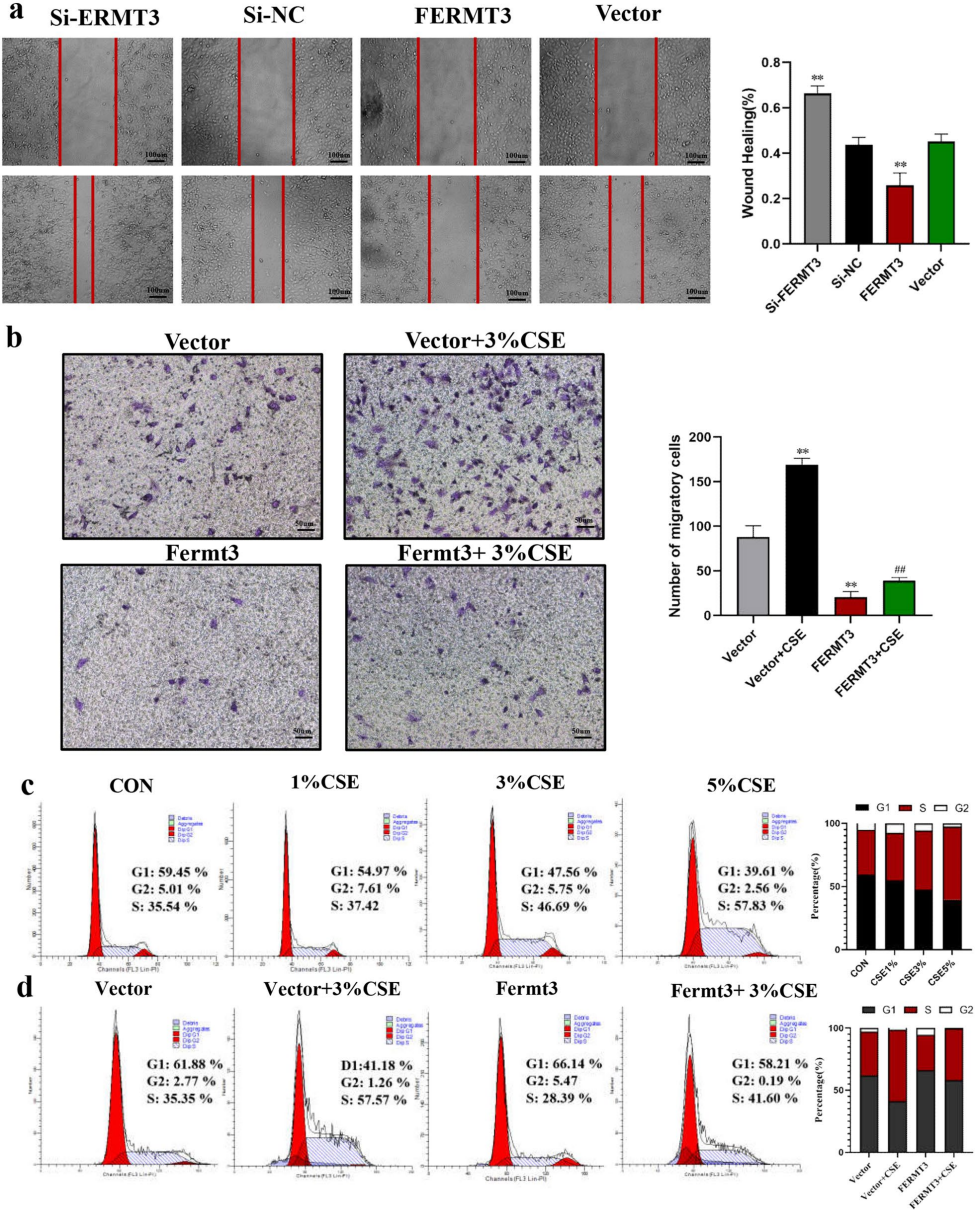
FERMT3 modulates cell migration and cell cycle upon CSE stimulation. **a** After transfection with si-NC, si-FERMT3, ferMT3 vector, or control vector for 24 h, the migration ability of A549 cells was detected by wound healing experiment. **P < 0.01, compared with Si-NC; ^##^P < 0.01, compared with control vector. **b**, **c** A549 cells were transfected with a control vector or FERMT3 vector then treated by 3% CSE for 24 h. **b** Transwell invasion assays of A549 cells (× 100 magnification). **c** Cell cycle of A549 cells assessed by flow cytometry. **P < 0.01, compared with control vector; ^##^P < 0.01, compared with control vector + 3% CSE. n = 3 per group

To explore the potential regulating role of FERMT3 in CSE-induced EMT, the A549 cell lines with knockdown or overexpression of FERMT3 were established by Si-FERMT3 transfection or FERMT3 vector, respectively. RT-PCR and western blot and confirmed knockdown or overexpression efficiency of FERMT3 in A549 cells (Additional file 4: Fig. S3a–Additional file 5: Fig. S4b). We next determined how FERMT3 affects EMT markers in A549 cells without CSE exposure. As shown in Fig. 5a, without CSE stimulation, knockdown of FERMT3 significantly reduced the protein expression of E-cadherin and increased the protein expression of Vimentin and Snail. On the contrary, the expression of E-cadherin was significantly increased in A549 cells transfected with the FERMT3 vector, while the expression of Vimentin and Snail was relatively low. Furthermore, A549 cells were transfected with FERMT3 vector and control vector and then treated by 3% CSE for 24 h. The results showed that CSE exposure significantly reduced E-cadherin levels, and significantly increased the expression levels of Vimentin and Snail (Fig. 4a). Moreover, FERMT3 overexpression significantly reverses the alterations of E-cadherin, Vimentin, and Snail in CSE-stimulated A549 cells (Fig. 4b), revealing that FERMT3 can reverse CSE-induced EMT in A549 cells.

Next, we observed the morphological changes of CSE-stimulated A549 cells after transfected with the control vector or FERMT3 vector. After exposure to CSE, the cells gradually displayed morphological appearances of mesenchymal cells. Moreover, cells transfected with the FERMT3 vector show a more epithelial-like morphology, suggesting that FERMT3 overexpression significantly reverses CSE-induced EMT in A549 cells (Fig. 4c).

### FERMT3 modulates cell migration and cell cycle upon CSE stimulation

Studies have indicated that the occurrence of EMT could affect the cell migration and cell cycle [20], we then evaluated whether FERMT3 could regulate CSE-induced cell migration and cell cycle. As shown in Fig. 5a, without CSE stimulation, wound healing assay Cell migration revealed that knockdown of FERMT3 significantly enhanced migration of A549 cells whereas FERMT3 overexpression significantly inhibited cell migration. Moreover, transwell migration assays revealed that CSE exposure significantly promoted cell migration ability, and overexpression of FERMT3 significantly reversed CSE-induced cell migration (Fig. 5b). Previous studies indicated that CSE exposure promoted cell cycle progression and accelerated the G1/S transition in HBE cells [21, 22]. In our work, A549 Cells were treated with 0, 1%, 3%, and 5% CSE for 24 h. Cell cycle results verified that CSE exposure promoted G1/S transition in A549 cells in a dose-dependent manner (Fig. 5c). However, pretreatment with FERMT3 vector transfection reversed this effect. FERMT3 overexpression blocked G1/S cell cycle transition, with the percentage of cells in the G1 and S phases recovering (Fig. 5d). Taken together, these results suggest that FERMT3 might be an important role in CSE-induced EMT in COPD.

### FERMT3 regulates CSE-induced EMT via Wnt/β-catenin signaling

**Fig. 6:**
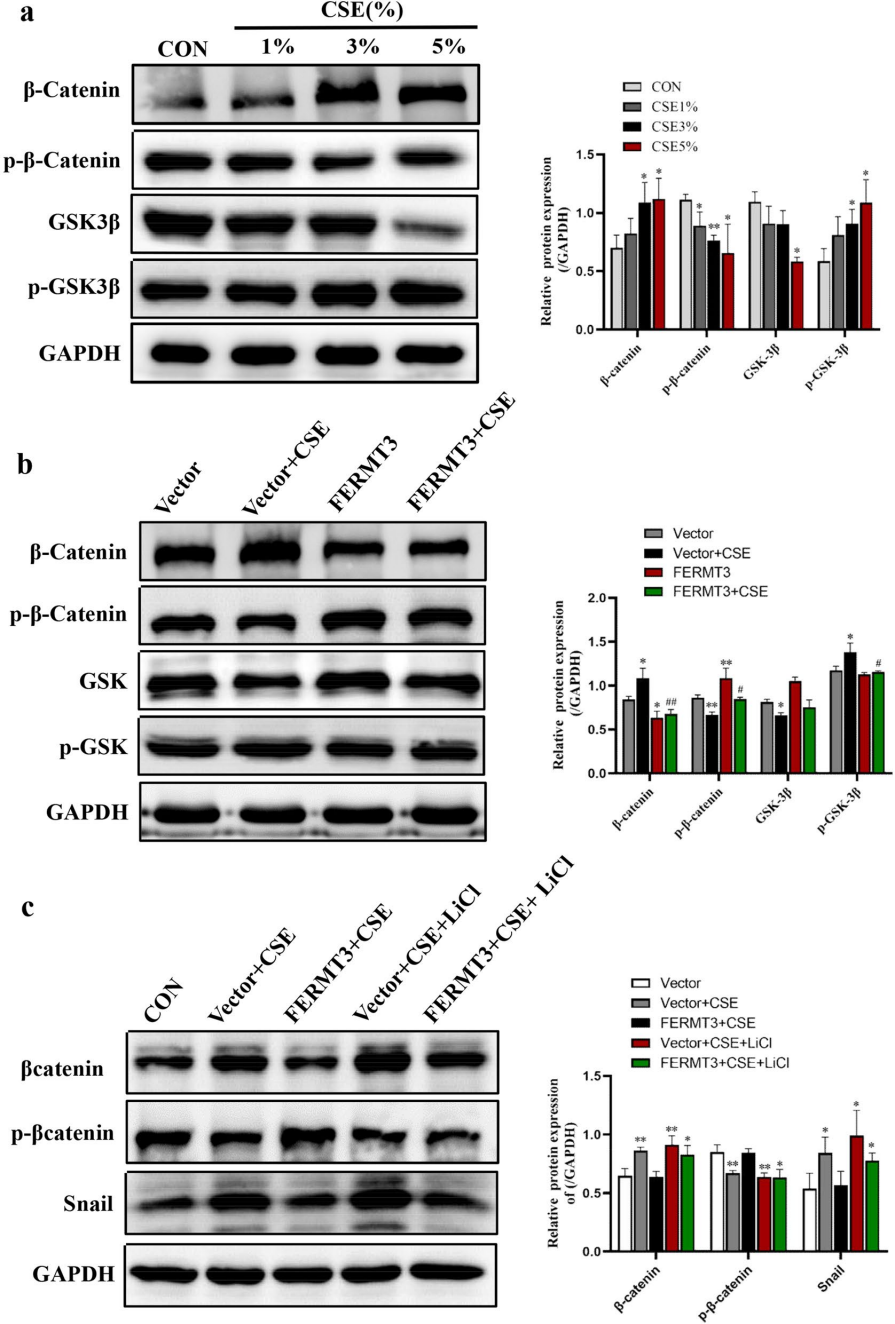
FERMT3 regulates CSE-induced EMT via Wnt/β-Catenin signaling. **a** β-catenin, p-β-catenin, GSK-3β and p-GSK-3β protein expression in A549 cells treated by CSE in a series of concentrations CSE for 24 h. n = 3 per group. *P < 0.05, **P < 0.01, compared with Control. **b** β-catenin, p-β-catenin, GSK-3β and p-GSK-3β protein expression in A549 cells transfected with control vector or FERMT3 vector then treated by 3% CSE for 24 h. *P < 0.01, **P < 0.01, compared with control vector; ^#^P < 0.05, ^##^P < 0.01, compared with control vector + 3% CSE. n = 3 per group. **c** β-catenin, p-β-catenin and Snail protein expression in A549 cells were transfected with control vector or FERMT3 vector, treated with 3% CSE for 24 h, and then treated with activator LiCl (20 mM) for 24 h. *P < 0.01, **P < 0.01, compared with control vector. n = 3 per group

We next hope to explore the mechanism involved in FERMT3 regulates the progression of EMT. Numerous studies reveal that the Wnt/β-catenin signaling plays a crucial role in airway remodeling and EMT in COPD [7, 23]. We first validated that CSE induced activation of the Wnt/β-catenin signaling. The data showed that 24 h CSE exposure (0, 1%, 3%, and 5%) resulted in β-catenin and p-GSK3β increased in a dose-dependent manner, and p-β-catenin and GSK3β reduced in a dose-dependent manner (Fig. 6a). While FERMT3 overexpression suppressed CSE-induced β-catenin and phosphorylation of GSK-3β and promotes β-catenin phosphorylation (Fig. 6b). These results indicated that FERMT3 may alleviate CSE-induced EMT through downregulation of the β-catenin pathway. To further confirm the role of the Wnt/β-catenin signaling pathway, A549 cells were transfected with a control vector or FERMT3 vector, treated with 3% CSE for 24 h, and then treated with activator LiCl (20 mM) for 24 h. As shown in Fig. 6c, the changes in EMT-inducing transcriptional factor Snail, β-catenin, p-β-catenin caused by FERMT3 overexpression could be partly reversed by LiCl. In addition, A549 cells were treated with activator LiCl for 24 h, PCR and western blot analysis showed FERMT3 expression has not changed significantly (Additional file 5: Fig. S4a, b). These observations suggested that FERMT3 is not downstream but possibly upstream of Wnt/β-catenin signaling. In summary, these data indicate that FERMT3 overexpression may inhibit the CSE-induced EMT via suppressing the Wnt/β-catenin signaling.

## Discussion

Cigarette smoke, as the most crucial risk factor in COPD and lung cancer, activates the EMT process, which contributes to the development and progression of COPD. In this study, we explored the potential role of FERMT3 in COPD and its underlying molecular mechanisms. According to GEO dataset analysis, FERMT3 expression in COPD smokers was lower than that in non-smokers or smokers, and FERMT3 was significantly down-regulated in lung tissues of COPD GOLD 4 patients compared with the control group. Furthermore, we observed that FERMT3 was downregulated in CS-exposed mice and CSE-exposed A549 cells. These results demonstrated that FERMT3 may be involved in COPD. Further studies indicated that overexpression of FERMT3 suppressed CSE-induced EMT in A549 cells. Moreover, the CSE-induced cell migration and cell cycle progression were reversed by FERMT3 overexpression. Mechanistically, CSE exposure resulted in Wnt/β-catenin signaling activation, which was suppressed by FERMT3 overexpression. Additionally, activator lithium chloride effectively rescued FERMT3-mediated inhibition of Wnt/β-catenin signaling.

Epithelial–mesenchymal transition (EMT) is a biological process in which differentiated epithelial cells lose polarity and acquire the motility and invasiveness characteristics of mesenchymal cells. EMT is broadly divided into three types, Type I occurs during embryogenesis, Type II in tissue repair and organ fibrosis, and Type III in epithelial malignancy. Both of the two latter types may be a link between COPD and Lung Cancer [24]. In addition, classical EMT is characterized by the loss of epithelial biomarkers (e.g., E-cadherin) and gain of biomarkers markers (e.g., Vimentin and Snail). EMT plays an important role in airway fibrosis and subsequent airflow obstruction in COPD and maybe a potential mechanism for the high prevalence of lung cancer in patients with COPD. Recent studies confirmed that cigarette smoking-induced EMT in human type II AEC cell line (A549) [18], the human bronchial epithelial cell line (16-HBE cells) [19, 25, 26], another human bronchial epithelial cell line BEAS-2B [22, 23], and primary human bronchial epithelial cell of smokers with COPD in vitro [27]. In the present study, we evaluated the effect of cigarette smoke on EMT in vivo and in vitro. The results confirmed that cigarette smoke-induced EMT in CS-exposure mice. Meanwhile, in vitro also demonstrated that EMT could be able to induce by CSE treatment in a dose-dependent manner in A549 cells. Therefore, EMT might be a novel target for the treatment of COPD.

Kindlin family (FERMT1, FERMT12, and FERMT13) are newly discovered adhesive proteins with a wide range of biological functions [11, 28]. FERMT3 is one of the Kindlin family members, involved in many important physiopathological processes [12, 13, 29]. Studies have shown that loss of Kindlin-3 increased mesenchymal stem cell proliferation [30] and FERMT3 was downregulated in several tumor types namely melanoma, breast, and lung cancers, and plays a tumor suppressor role in solid cancer by regulating cell adhesion, migration and invasion [10]. However, the role of FERMT3 in COPD, including EMT, has not so far been defined well. In our study, we found the expression of FERMT3 was downregulated in COPD patients according to GEO datasets analysis. Furthermore, we reported for the first time that the expression of FERMT3 was downregulated in CS-exposure mice and observed in a dose- and time-dependent manner downregulation of FERMT3 in A549 cells after CSE treatment. Then we demonstrated that overexpression of FERMT3 inhibited CSE-induced EMT in A549 cells. In addition, overexpression of FERMT3 reversed CSE-induced cell migration and cell cycle progression. Taken together, these data indicate that FERMT3 may be involved in the occurrence and development of COPD. However, further in-depth studies are required to verify this point.

Although the mechanism of EMT in COPD has not been fully elucidated, the Wnt/β-catenin signaling, the transforming growth factor-β (TGF-β)/Smad signaling are believed to be involved in EMT [5]. The Wnt/β-catenin signaling has been involved in the process of EMT [31], an intermediate between smoking and airway remodeling, and indeed lung cancer [6, 26, 32]. Increasing evidence indicated that the Wnt/β-catenin signaling is upregulated in smokers and COPD, and Wnt/β-catenin signaling is closely associated with EMT activity and airway obstruction [7, 33]. Zou et al. found that cigarette smoke and nicotine-induced EMT via Wnt/β-catenin signaling activation in HBE cells [26]. Recently studies have shown that the Kindlin family can moderately regulate the occurrence and development of many diseases involved in the Wnt/β-catenin signaling [34–37]. In addition, studies have also found that members of the Kindlin family are involved in the regulation of EMT in different diseases [38–40]. A previous study reported that FERMT3 contributed to the occurrence of glioblastoma through activation of the Wnt pathway [37]. Regarding the underlying mechanism of FERMT3 on the above biological function, we explored whether FERMT3 regulates CSE-induced EMT through the Wnt/β-catenin signaling. In our work, CSE exposure attenuated activation of Wnt/β-catenin signaling and the activation effect could be reversed by FERMT3 overexpression. To further verify the role of the Wnt/β-catenin pathway, activator lithium chloride was added. The results presented that LICL could partially reverse the inhibition of overexpression of FERMT3 on β-catenin, p-β-catenin, and Snail expression. Altogether, our study suggests that FERMT3 overexpression may inhibit CSE-induced EMT in A549 cells by inhibiting the Wnt/β-catenin signaling.

Meanwhile, this research exists some limitations. First, A549 cells were used as ATII cells in this study. A549 is derived from a human lung adenocarcinoma cell line.

However, studies have shown that A549 cells have the characteristics of type II alveolar epithelial cells and is therefore widely used in ATII cell model and EMT studies [41–43]. Also, the role that FERMT3 modulates cigarette smoke-induced EMT plays in airway remodeling, fibrosis, and malignant transformation may require further exploration. In addition, how FERMT3 regulates Wnt/β-catenin signaling and whether FERMT3 affects other involving mechanisms needs further investigation.

## Conclusion

**Fig. 7:**
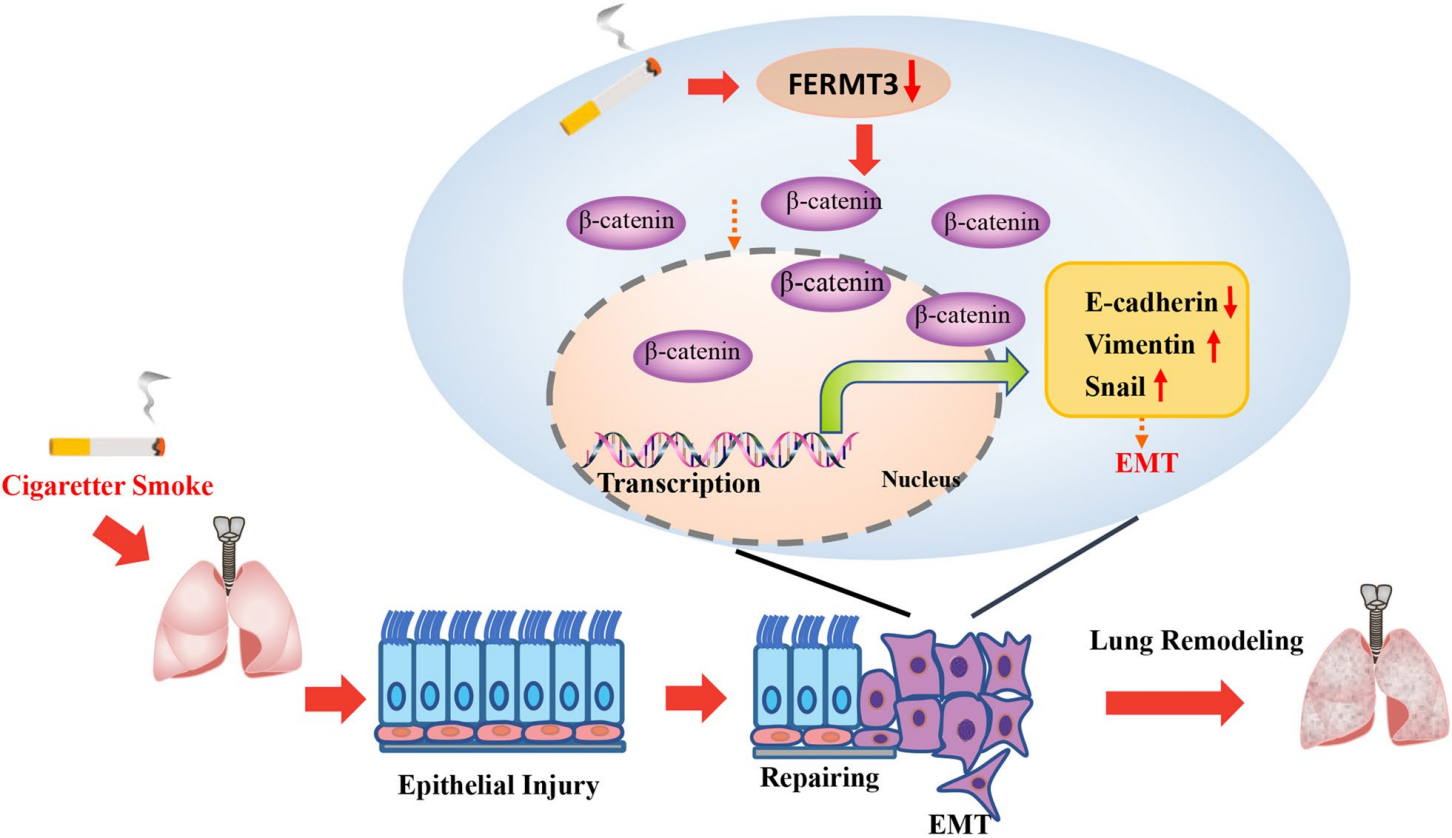
Schematic representation for plausible mechanism showing that FERMT3 mediates cigarette smoke extract-induced epithelial–mesenchymal transition in alveolar epithelial cells via Wnt/β-Catenin signaling

In summary, we demonstrated that FERMT3 expression was downregulated in COPD patients, CS exposed mice, and CES-induced A549 cells. Our data suggested that FERMT3 could mediate CSE-induced epithelial–mesenchymal transition via Wnt/β-catenin signaling (Fig. 7). Our study provides a new perspective for understanding EMT and suggests that FERMT3 is a potential target for further understanding of the mechanisms of COPD and lung cancer.

## Supplementary Information

**Additional file 1: Table S1.** Characteristics of study individuals (GSE13896). **Table S2.** Characteristics of study individuals (GSE130928). **Table S3.** Characteristics of study individuals (GSE51052).

**Additional file 2: Fig. S1.** Evaluation of chronic CS-induced COPD mouse model. a Mice Lung histology from mice exposed to CS or control air was analyzed via H&E staining (× 100 magnification). n = 6 mice/per group. b–d Pulmonary function measurement in mice model. FEV0.2%: the ratios of forced expiratory volume (FEV) at 0.2 s (FEV0.2) to forced vital capacity; RI: Inspiratory resistance; FRC: functional residual capacity. *P < 0.05, **P < 0.01, compared with Control.

**Additional file 3: Fig. S2.** Effects of CSE on cell viability in A549 cells and HBE cells. a CCK-8 assay showed the effects of different concentrations of CSE (0%, 1%, 3%, 5%, 7% and 10%) at different times (0, 12, 24, and 48 h) on A549 cell viability. b CCK-8 assay showed the effects of different concentrations of CSE (0%, 1%, 2%, 3%, 5% and 10%) at different times (0, 12, 24, and 48 h) on HBE cell viability. n = 5 per group.

**Additional file 4: Fig. S3.** Analysis of FERMT3 transfection efficiency. A549 cells were transfected withSi-FERMT3 transfection, Si-NC, FERMT3 vector or control vector for 24 h. a The transfection efficiency was detected by RT-PCR. **P < 0.01, compared with Control. n = 3 per group. b The transfection efficiency was detected by western blot. **P < 0.01, compared with Si-NC; P < 0.01, compared with control vector. n = 3 per group.

**Additional file 5: Fig. S4.** Evaluating the effect of LiCl on FERMT3 in A549 cells. A549 cells were treated with activator LiCl for 24 h. The effect of LiCl on FERMT3 was detected by a PCR and b western blot.

## Data Availability

Three datasets (GSE130928, GSE13896 and GSE151052) analyzed during the current study are available in the GEO, https://www.ncbi.nlm.nih.gov/geo/. The data during the study are available from the corresponding author on reasonable request.

## Abbreviations

COPD: Chronic obstructive pulmonary disease
EMT: Epithelial–mesenchymal transition
GEO: Gene Expression Omnibus
TGF-β: Transforming growth factor-β
CS: Cigarette smoke
CSE: Cigarette smoke extract
FEV: Forced expiratory volume
FRC: Functional residual capacity
RI: Inspiratory resistance
CCK-8: Cell Counting Kit-8
H&E: Hematoxylin–eosin staining
