# Supplementary material for: FERMT3 mediates cigarette smoke-induced epithelial–mesenchymal transition through Wnt/β-catenin signaling
Source: Respir Res. 2021 Nov 6;22:286. doi: 10.1186/s12931-021-01881-y (PMC8571878; doi:10.1186/s12931-021-01881-y)
Supplement: Supplementary file 1 — Additional file 1: Table S1. Characteristics of study individuals (GSE13896). Table S2. Characteristics of study individuals (GSE130928). Table S3. Characteristics of study individuals (GSE51052). [file 12931_2021_1881_MOESM1_ESM.docx]

Table S1. Characteristics of study individuals (GSE13896)

| Characteristic | All | Non-smoker | Smokers | COPD-smokers |
| --- | --- | --- | --- | --- |
| n (%) | 70 | 24 (34.3) | 34 (48.6) | 12 (17.1) |
| Females, n (%) | 17 (24.3) | 6 (8.6) | 9 (12.8) | 2 (2.9) |
| Age, years | 43(35,48) | 40(35,47) | 42(39,47) | 51(27,71) |
| Smoking pack-years |  | NA | 27(16-40) | 51(28,75) |

Table S2. Characteristics of study individuals (GSE130928)

| Characteristic | All | Non-smoker | Smokers | COPD-smokers |
| --- | --- | --- | --- | --- |
| n (%) | 88 | 24 (27.3) | 42 (48.7) | 22 (25) |
| Females, n (%) | 22 (25) | 6 (6.8) | 12 (13.6) | 4 (4.6) |
| Age, years | 45 (38-49) | 40 (35-47) | 43 (40-47) | 54 (58-46) |
| Smoking pack-years | 24(0-33) | NA | 26 (16-36) | 43 (24,51) |

Table S3 Characteristics of study individuals (GSE51052)

| Characteristic | All | Control | COPD |
| --- | --- | --- | --- |
| n (%) | 15 | 5 | 10 |
| Tissue | NA | unused donor control lung tissue | transplanted lung tissue |
| Age, years | 45（38-49） | 60 (42,77) | 55 (39-77) |
| GOLD grades | 24(0-33) | NA | GOLD 4 |

Continuous variables are described by median values (25th percentile to 75th percentile). Dichotomous variables are presented as total numbers (%). NA = data not available.
