# Supplementary figures and images for: FERMT3 mediates cigarette smoke-induced epithelial–mesenchymal transition through Wnt/β-catenin signaling
Source: Respir Res. 2021 Nov 6;22:286. doi: 10.1186/s12931-021-01881-y (PMC8571878; doi:10.1186/s12931-021-01881-y)

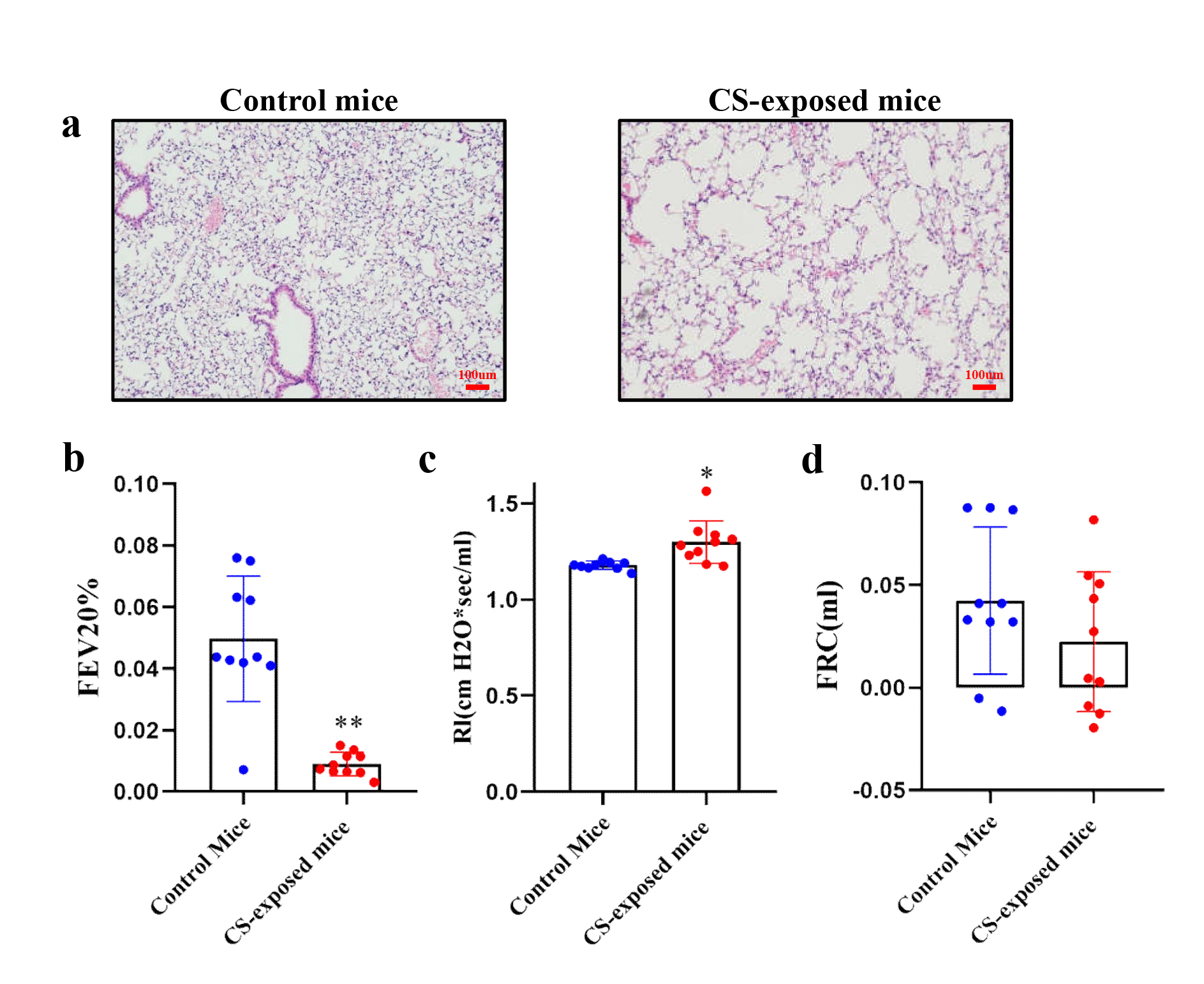

Supplement: Supplementary file 2 — Additional file 2: Fig. S1. Evaluation of chronic CS-induced COPD mouse model. a Mice Lung histology from mice exposed to CS or control air was analyzed via H&E staining (× 100 magnification). n = 6 mice/per group. b–d Pulmonary function measurement in mice model. FEV0.2%: the ratios of forced expiratory volume (FEV) at 0.2 s (FEV0.2) to forced vital capacity; RI: Inspiratory resistance; FRC: functional residual capacity. *P < 0.05, **P < 0.01, compared with Control. [file 12931_2021_1881_MOESM2_ESM.jpg]

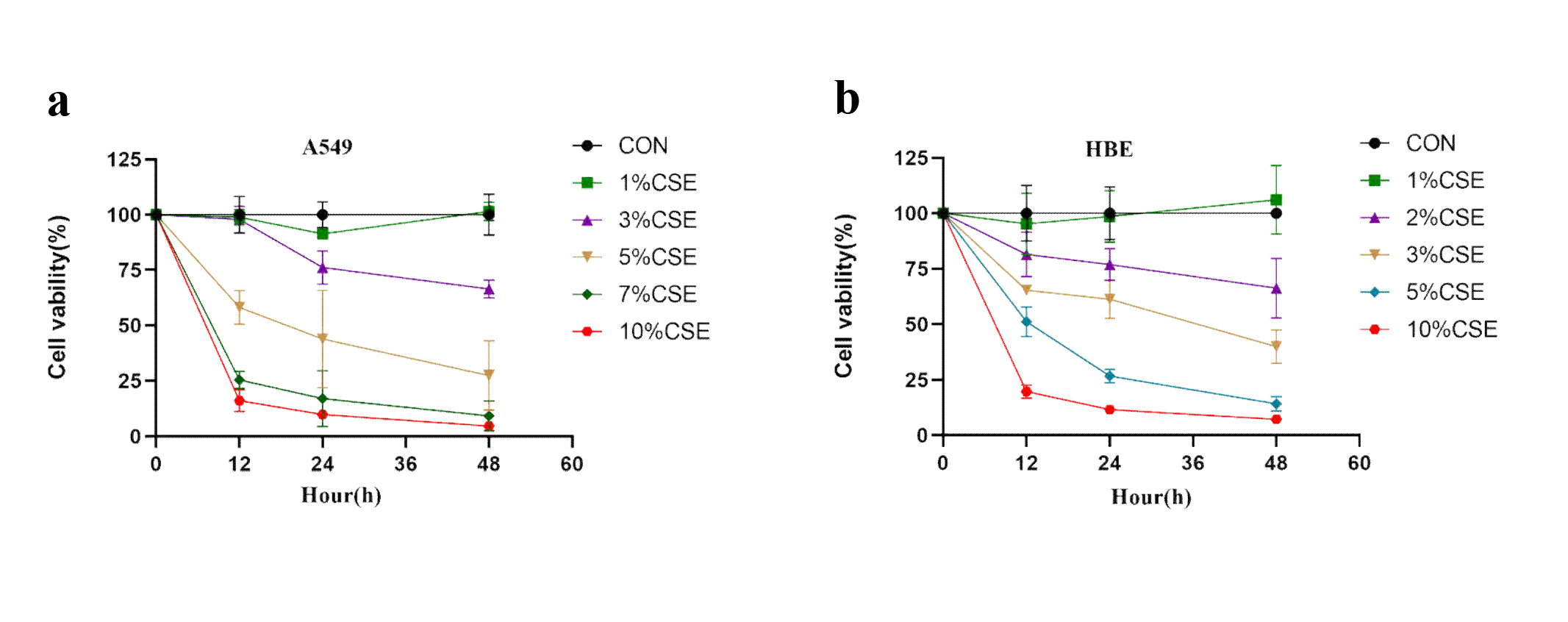

Supplement: Supplementary file 3 — Additional file 3: Fig. S2. Effects of CSE on cell viability in A549 cells and HBE cells. a CCK-8 assay showed the effects of different concentrations of CSE (0%, 1%, 3%, 5%, 7% and 10%) at different times (0, 12, 24, and 48 h) on A549 cell viability. b CCK-8 assay showed the effects of different concentrations of CSE (0%, 1%, 2%, 3%, 5% and 10%) at different times (0, 12, 24, and 48 h) on HBE cell viability. n = 5 per group. [file 12931_2021_1881_MOESM3_ESM.jpg]

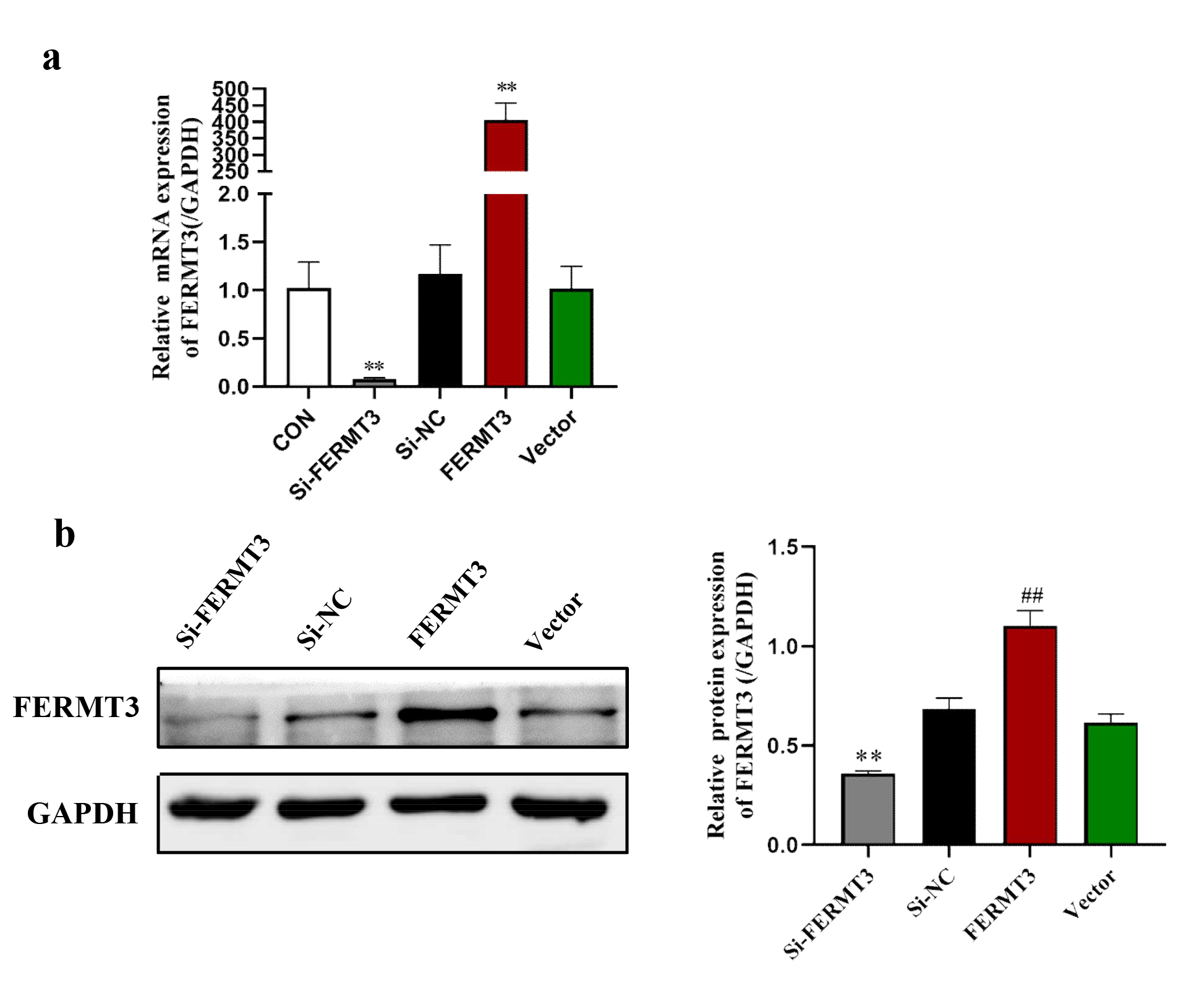

Supplement: Supplementary file 4 — Additional file 4: Fig. S3. Analysis of FERMT3 transfection efficiency. A549 cells were transfected withSi-FERMT3 transfection, Si-NC, FERMT3 vector or control vector for 24 h. a The transfection efficiency was detected by RT-PCR. **P < 0.01, compared with Control. n = 3 per group. b The transfection efficiency was detected by western blot. **P < 0.01, compared with Si-NC; P < 0.01, compared with control vector. n = 3 per group. [file 12931_2021_1881_MOESM4_ESM.jpg]

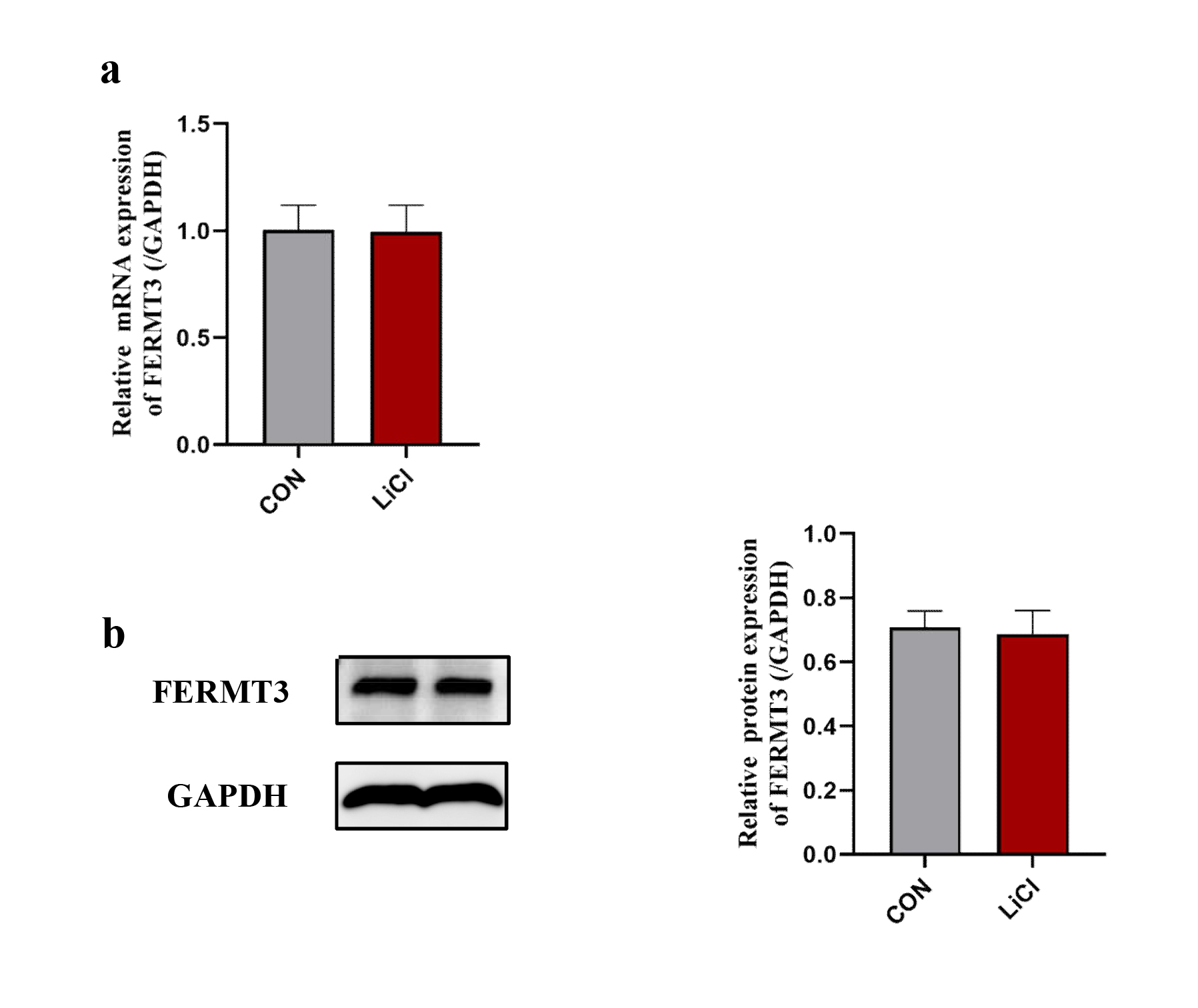

Supplement: Supplementary file 5 — Additional file 5: Fig. S4. Evaluating the effect of LiCl on FERMT3 in A549 cells. A549 cells were treated with activator LiCl for 24 h. The effect of LiCl on FERMT3 was detected by a PCR and b western blot. [file 12931_2021_1881_MOESM5_ESM.jpg]
